# Supplementary material for: Differences in Tumor Growth and Differentiation in NSG and Humanized-BLT Mice; Analysis of Human vs. Humanized-BLT-Derived NK Expansion and Functions
Source: Cancers (Basel). 2022 Dec 24;15(1):112. doi: 10.3390/cancers15010112 (PMC9817973; doi:10.3390/cancers15010112)
Supplement: Supplementary file 1 [file cancers-15-00112-s001.zip › cancers-2068617-supplementary.pdf]

Supplementary data

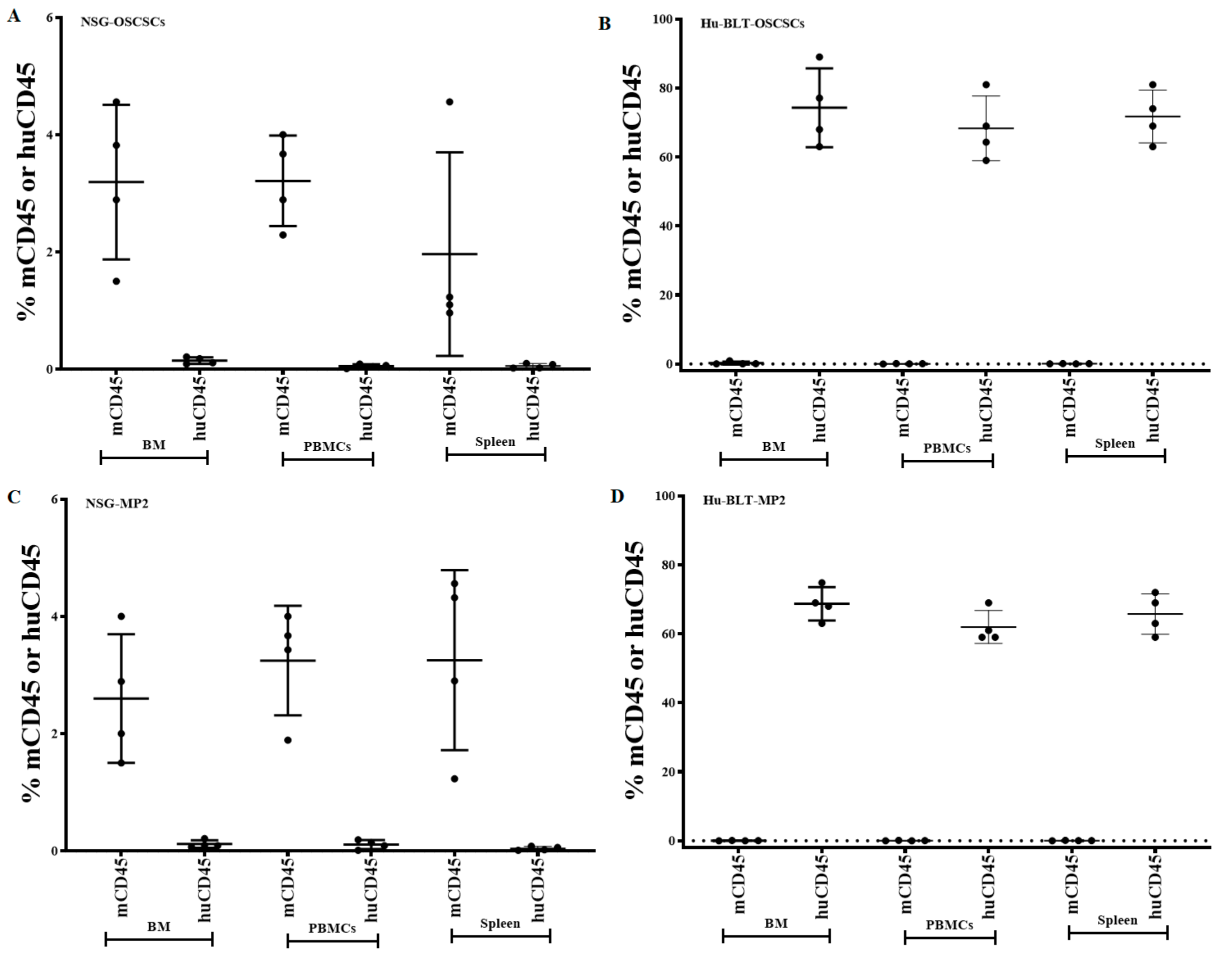

Figure S1. Immune cells reconstitution of NSG and hu-BLT mice.

NSG and Hu-BLT mice were surgically implanted with 1 X 10^6 tumor cells either in pancreas or oral cavity, and disease progression was monitored for four weeks. After euthanasia, tissues were resected to obtain single-cells. Percentages of human and mice CD45+ immune cells were determined in single cells isolated from BM, PBMCs and spleen (n=4) **(A, B, C, D)**.

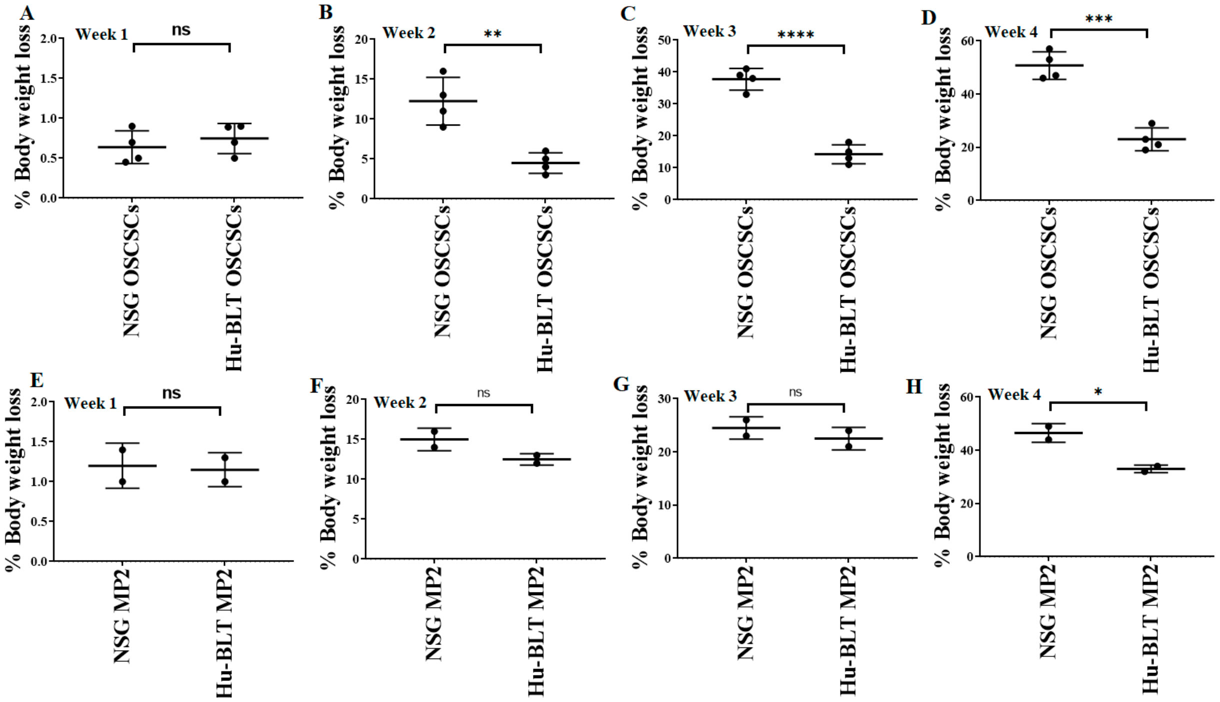

Figure S2. Higher body weight loss in NSG mice in comparison to hu-BLT mice after surgical implantation of OSCSCs or MP2 tumors. NSG and Hu-BLT mice were surgically implanted with 1 X 10^6 tumor cells in the oral cavity **(A,** n=4**)**, or pancreas **(B**, n=2**)**, and rate of percentages of body weight loss were determined on weeks 1, 2, 3 and 4.

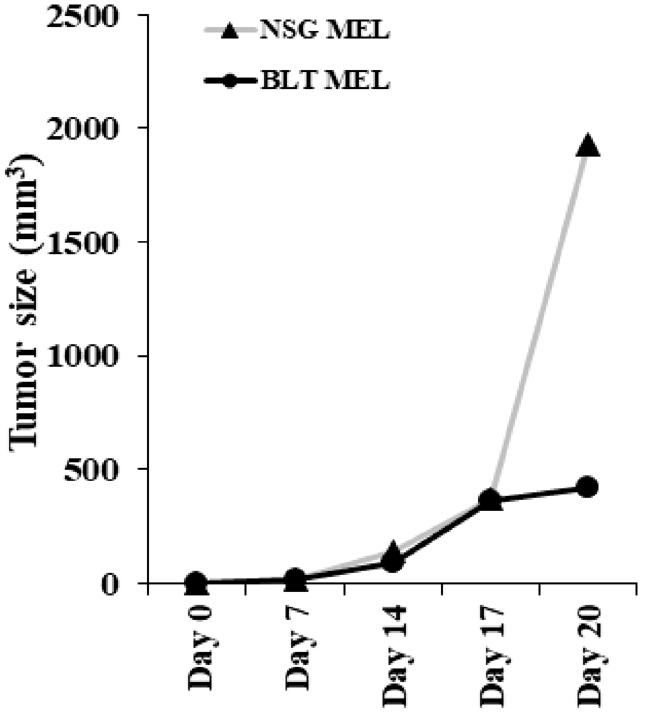

Figure S3: higher tumor growth in NSG mice in comparison to hu-BLT mice after orthotopic implantation of melanoma tumors.

NSG and Hu-BLT mice were implanted with $1X10^6$ tumor cells subcutaneusly. Tumor size was measured using caliper on days shown in the figure.

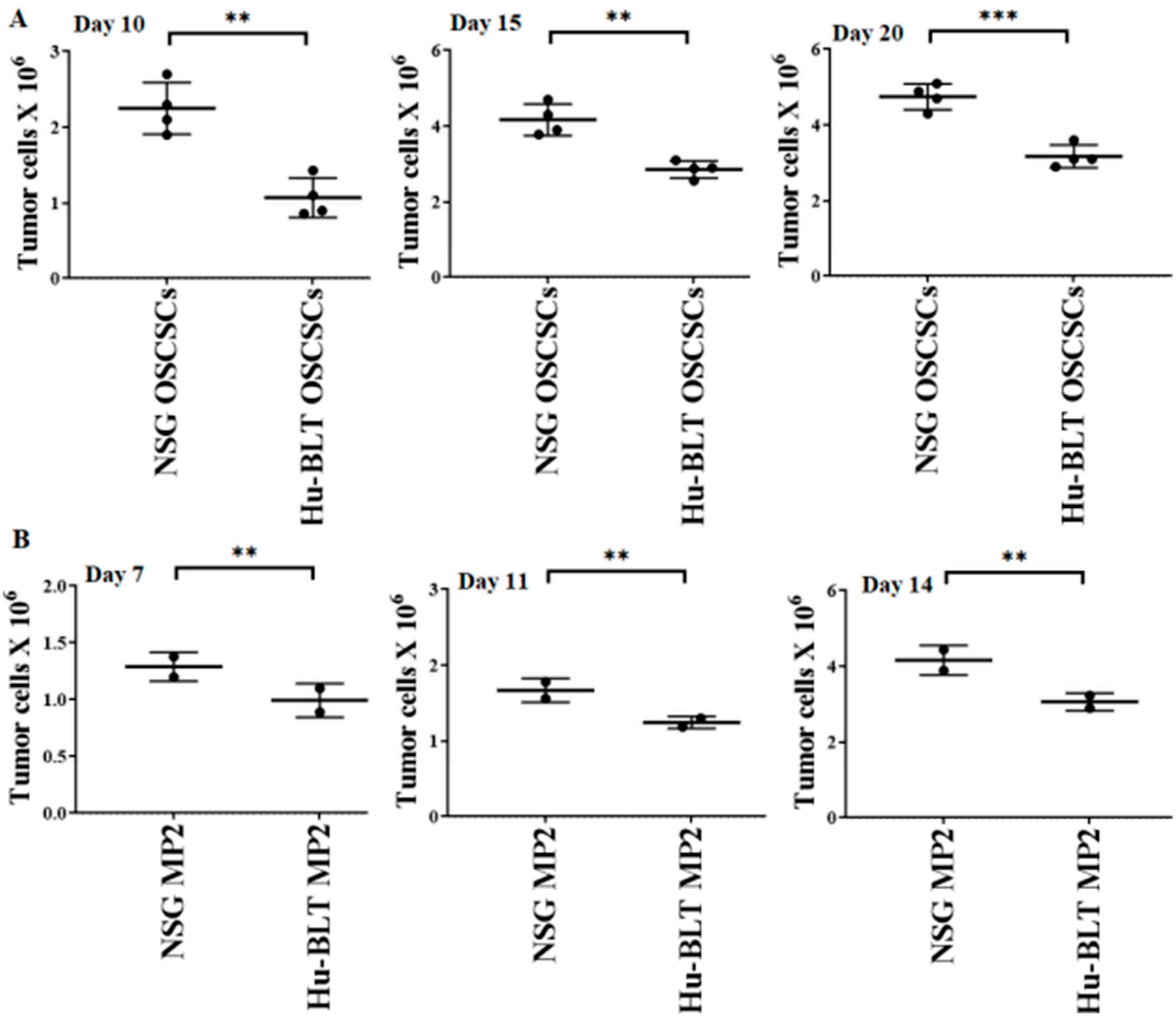

Figure S4: Significantly higher tumor growth in cultures of tumors resected from NSG mice in comparison to hu-BLT mice.

NSG and Hu-BLT mice were implanted with $1X10^6$ tumor cells in the oral cavity or pancreas. On week 4, mice were euthanatized, tumors were resected and were cultured (1×10^6 cells/ml). Tumor cells were counted on days as shown in the figures.

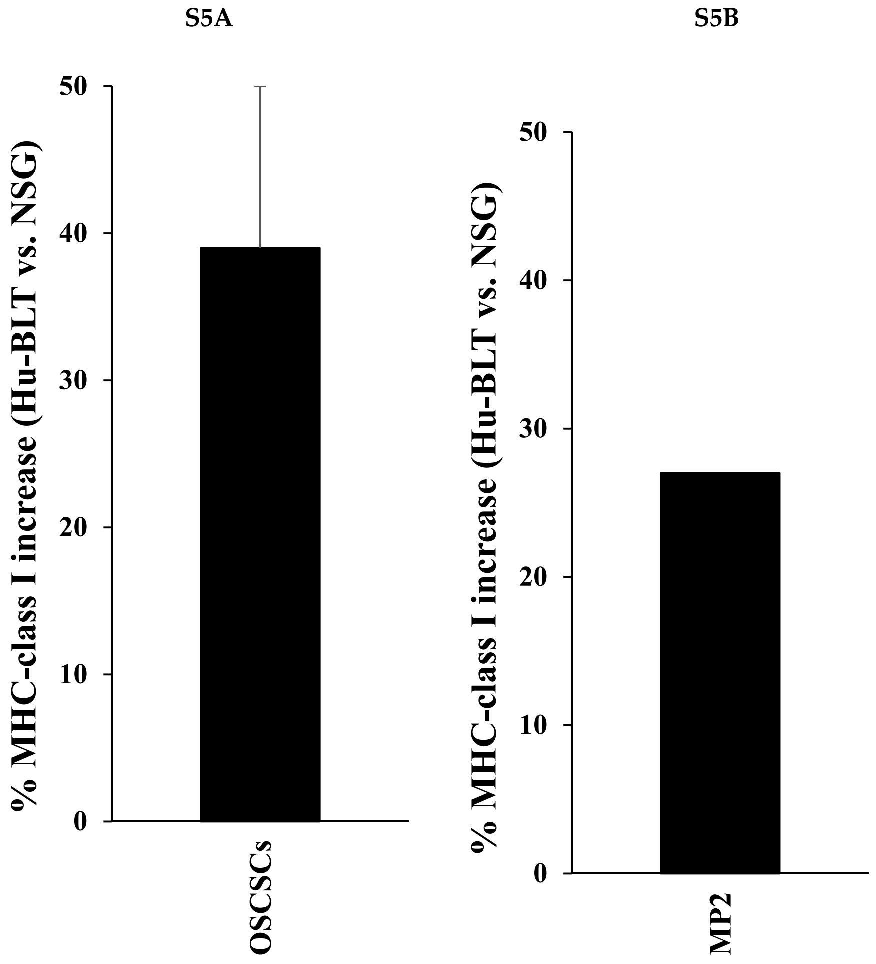

Figure S5: Surface expression levels of MHC-class I on tumors isolated from NSG and hu-BLT mice.

NSG and Hu-BLT mice were implanted with $1X10^6$ tumor cells in the oral cavity **(A)** and pancreas **(B)**. On week 4, mice were euthanatized, tumors were resected, and were cultured ($1X10^6$ cells/ml) for 7 days. The surface expressions of MHC-class I were determined on day 7 tumor cultures, and fold percentage increase on tumors dissected from hu-BLT mice vs. those dissected from NSG mice was determined (n=3).
